# A Comparison of Thresholding Methods for Forensic Reconstruction Studies Using Fluorescent Powder Proxies for Trace Materials†

**DOI:** 10.1111/1556-4029.13938

**Published:** 2018-10-25

**Authors:** Emma A. Levin, Ruth M. Morgan, Lewis D. Griffin, Vivienne J. Jones

**Affiliations:** ^1^ Centre for the Forensic Sciences University College London 35 Tavistock Square London WC1H 9EZ U.K; ^2^ Department of Security and Crime Science University College London 35 Tavistock Square London WC1H 9EZ U.K; ^3^ Environmental Change Research Centre Department of Geography University College London Pearson Building, Gower Street London WC1E 6BT U.K; ^4^ Deparment of Computer Science University College London Gower Street London WC1E 6BT UK

**Keywords:** forensic science, trace evidence, transfer and persistence, ultraviolet, fluorescence, image processing, segmentation, thresholding

## Abstract

Image segmentation is a fundamental precursor to quantitative image analysis. At present, no standardised methodology exists for segmenting images of fluorescent proxies for trace evidence. Experiments evaluated (i) whether manual segmentation is reproducible within and between examiners (with three participants repeatedly tracing three images) (ii) whether manually defining a threshold level offers accurate and reproducible results (with 20 examiners segmenting 10 images), and (iii) whether a global thresholding algorithm might perform with similar accuracy, while offering improved reproducibility and efficiency (16 algorithms tested). Statistically significant differences were seen between examiners’ traced outputs. Manually thresholding produced good accuracy on average (within ±1% of the expected values), but poor reproducibility (with multiple outliers). Three algorithms (Yen, MaxEntropy, and RenyiEntropy) offered similar accuracy, with improved reproducibility and efficiency. Together, these findings suggest that appropriate algorithms could perform thresholding tasks as part of a robust workflow for reconstruction studies employing fluorescent proxies for trace evidence.

Powder which fluoresces under ultraviolet light has been successfully employed as a proxy for microscopic trace evidence in experiments exploring the transfer and persistence of particulates in forensically relevant scenarios 1, 2, 3. With a diameter of approximately 15 μm 2, fluorescent powder has been used to represent diverse evidence types, including silt‐sized elements of soil 1, 4 and pollen grains 5. Utilising a fluorescent proxy can allow for the rapid generation of data, since sampling can be nondestructive (photographic) rather than destructive (involving the physical or chemical processing of samples) 2. Accordingly, such studies can help to expand our understanding of trace evidence dynamics and, therefore, inform the nuanced interpretation of evidence within casework scenarios, a current priority for research within the forensic sciences 6.

Studies which utilise fluorescent proxies tend to follow similar methodologies; the fluorescent material is introduced to a surface, imaged repeatedly during the experiment, and the resultant images are segmented and analysed. At present, there does not appear to be a standardised methodology for segmenting images in the context of persistence studies which employ a fluorescent proxy, but most appear to involve a form of thresholding (Table 1; (e.g., 1, 3, 4, 5, 7). Where it is explicit that thresholding has been used to segment the images (e.g., 7) it is not readily apparent whether the threshold value was manually defined or calculated with an algorithm (Table 1).

**Table 1:** Examples of the methodologies used to process imagery from persistence studies which employ a fluorescent proxy for trace evidence

Year	Paper Title	Purpose of the UV	Image Processing and Analysis Method (Verbatim)
2006	The transfer and persistence of trace particulates: experimental studies using clothing fabrics	To emulate lighter flint particles 1	“photographs were pixelated using Corel Photo‐Paint 9 and the number of particles in each image were computed (as a function of pixel brightness)” (Bull et al. 1: 191)
2009	The Forensic Analysis of Sediments Recovered from Footwear	To explore the movement of silt‐sized particles 4	“This digital image was then pixelated in IDRISI to provide an indication of the amount of silt‐sized material remaining on the sole.” (Morgan et al. 4: 9)
2012	Multiple transfers of particulates and their dissemination within contact networks	To act as a proxy for particulate trace evidence while investigating multiple transfers 3	“The presence of UV powder on the stub was […] quantified using an image rasterisation technique in MATLAB which was specifically adapted for the specifications of this study from Bull et al.[2006]” (French et al. 3: 34)
2013	The recovery of pollen evidence from documents and its forensic implications	As a proxy for pollen on the surface of documents 5	“The digital images taken of each experiment were imported into Coral Photo Paint 11 [sic]. The images were graphically enhanced, pixelated and then five sections of 32 × 7 pixels were counted” (Morgan et al. 5: 377)
2017	Tracers as invisible evidence—The transfer and persistence of flock fibres during a car exchange	UV flock fibres as a tracer 7	“A Matlab (version R2012b) algorithm was created to enable fast automated counting of flock fibres on pictures. The individual pictures were loaded into Matlab and processed automatically. Firstly, the original RGB (red, green, and blue) colour images were converted to a grey value image by extracting the green channel. Subsequently, the foreground (i.e., the flock fibres) was separated from the background (i.e., the target materials) by thresholding. A region of interest (ROI) was selected as well. Next, the fibres were counted. As a result of the varying illumination conditions, the size of one fibre (in pixels) was not the same on all pictures and therefore had to be estimated for each image first. Subsequently, adding up all the foreground pixels and dividing them by the estimated number of pixels per fibre, yielded the amount of fibres on an image.” (Slot et al. 7: 181)

Thresholding is one of the simplest methods of image segmentation; a threshold value is defined for an attribute of each pixel (e.g., brightness or luminance levels), and every pixel's brightness value is reassigned to either black or white depending on whether the original value of the pixel fell above or below the threshold 8, 9. It has been firmly established within the fields of computer science and machine vision that different thresholding algorithms are appropriate for different contexts, and treating the same input image with different algorithms can result in very different outputs (8, 10, 11; Fig. 1). It has also been suggested that manually defining a threshold level may be unreliable, and therefore problematic as part of a reproducible workflow 12, 13, 14.

**Figure 1:**
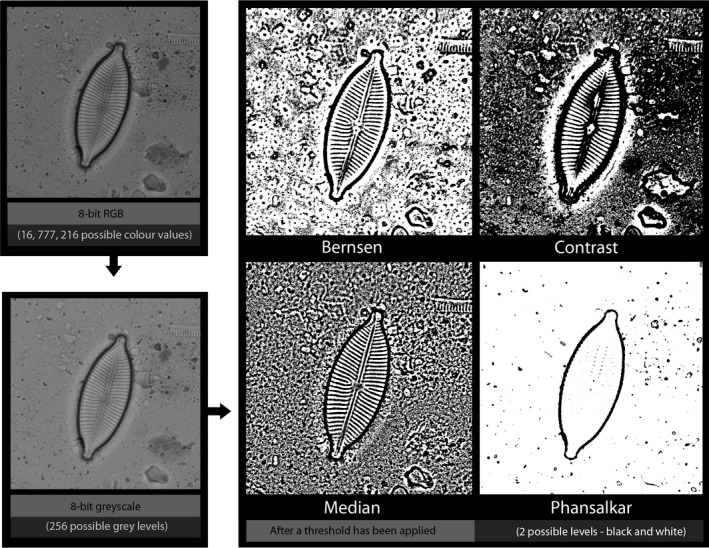
The results of applying four different local thresholding algorithms to the same input image (in this case a diatom, a form of environmental trace evidence). Algorithms were applied in ImageJ, with a radius of 15 pixels (from L‐R, Bernsen, Contrast, Median, and Phansalkar).

Accordingly, the aim of this paper was to evaluate the performance of different methods of thresholding upon the types of images likely to be encountered in the course of a persistence study employing a fluorescent proxy. Experiments were undertaken to explore the concerns that segmentation methods which involve manual input might be to a certain extent observer‐dependent and to explore whether different methods of thresholding were capable of segmenting the images with fidelity. The overarching aim was to establish whether one of the tested methods might be most suitable for use with this type of imagery, and particularly, for future experiments, whether a method would enable rapid and accurate processing of very large numbers of images.

Three experiments were undertaken, with the following aims:

1. Experiment One: To explore the extent to which the results of manual segmentation (i.e., tracing) are reproducible within and between examiners
2. Experiment Two: To explore the extent to which the results of segmentation by thresholding, where the value of the threshold is manually defined, are both reproducible within and between examiners and accurate compared to the original image
3. Experiment Three: To evaluate whether a global thresholding algorithm could segment the images with similar accuracy to the manual methods of segmentation, while offering improved reproducibility and efficiency

It can be noted that three aspects of performance are considered within these experiments: accuracy (the extent to which the produced segmentation was concordant with the expected segmentation), reproducibility (the extent to which the results of the technique would be reproducible if the segmentation were repeated), and efficiency (the amount of time required to employ the segmentation method (see 15).

## Evaluating Accuracy

Manual segmentation (tracing) is generally presented as the “gold standard” of image segmentation and, assumed to reflect the original image with fidelity, is often used as a stand‐in for a ground truth when evaluating the performance of novel segmentation methods 12, 15, 16, 17. In order for a segmentation method to be considered viable for future work, it should deliver good levels of accuracy (i.e., one should aim for fidelity to the original image).

## Evaluating Reproducibility

The second aspect of performance considered was the reproducibility of each segmentation technique (i.e., the extent to which measurements were reliable). While an algorithm should always return the same output from the same input (introducing deterministic error), manual processes may introduce both stochastic and deterministic error and it is entirely possible that providing an examiner with the same input image repeatedly, or providing multiple examiners with the same input image, might result in dissimilar outputs. This concept has been explored to some extent within the forensic sciences in the context of decision‐making under conditions of uncertainty, with studies evaluating the extent to which examiners consistently reach the same conclusions when visually examining ambiguous marks 18, 19. In order for a segmentation method to be considered viable for use in future experiments, it should produce similar, if not identical, results when used repeatedly or by different examiners.

## Evaluating Efficiency

Given that the time required to conduct visual data analysis can place a ceiling on the amount of data that can be collected over the course of experiments 20, it would be useful to arrive at a segmentation method that is not only comparatively accurate and comparatively reproducible, but also comparatively rapid (see also 15). Accordingly, this paper also intended to evaluate, on the basis of time, whether it would be viable to employ each segmentation method upon a large set of images.

The overall aim, therefore, was to identify a method of segmentation that could be used as part of a robust workflow for the analysis of ultraviolet fluorescence images in the context of future persistence studies.

## Materials

### Images

**Figure 2:**
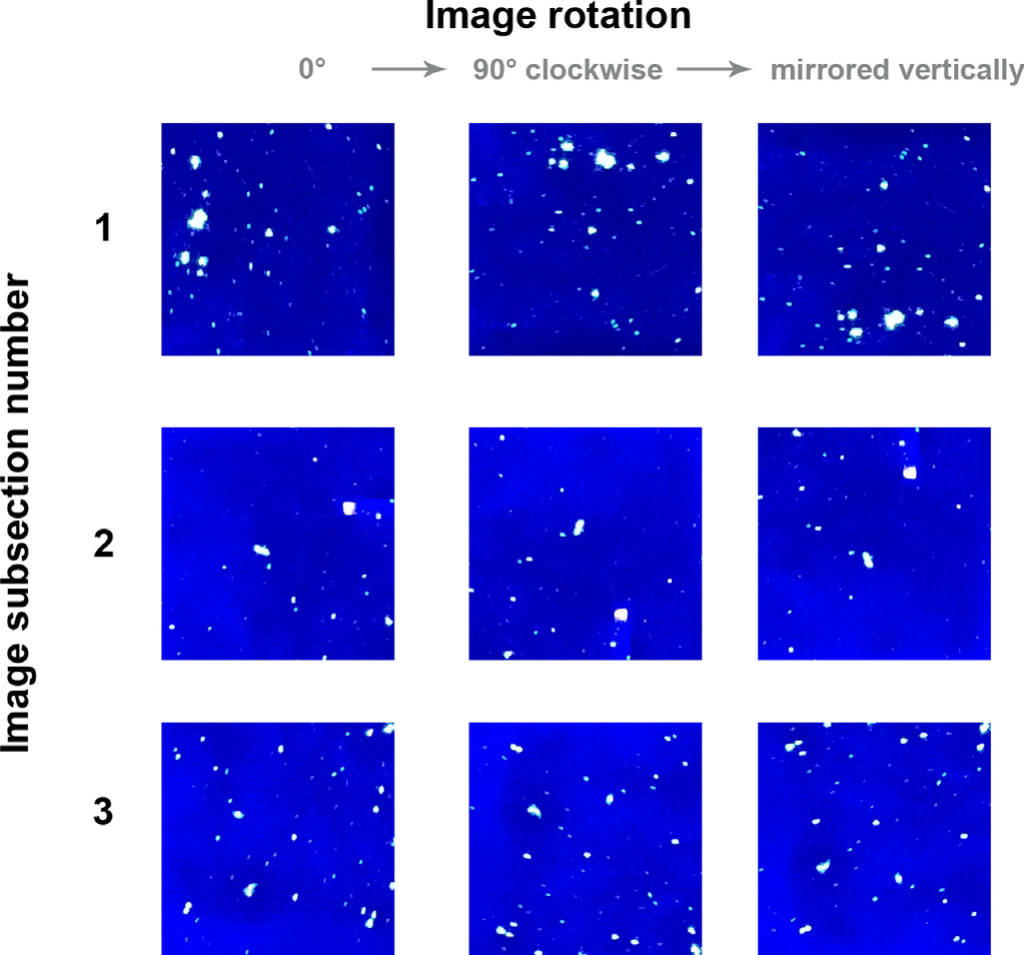
The images and rotations used in Experiment 1. [Color figure can be viewed at http://wileyonlinelibrary.com]

**Figure 3:**
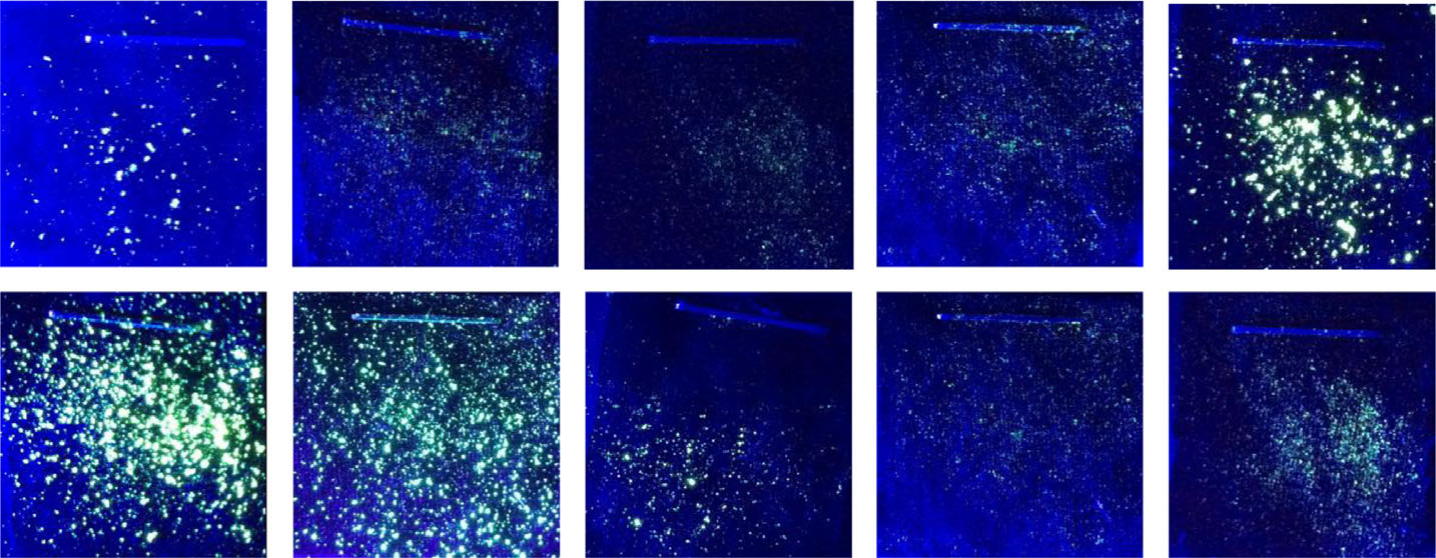
The images to which thresholds were applied in Experiments 2 and 3. [Color figure can be viewed at http://wileyonlinelibrary.com]

Images were generated during a persistence study where fluorescent powder with a median diameter of approximately 15 μm had been transferred to swatches of black cotton by flicking with a stiff‐bristled paintbrush, after 2. Ultraviolet illumination was provided with a 385 nm torch, and imaging followed the procedure outlined in 3, with samples housed in a purpose‐built UV‐dark box which excluded visible light and held the torch and camera lens a fixed distance from the samples. Images were acquired as 8‐bit red, green, and blue (RGB) colour (see Figs 2 and 3). Each swatch was composed of multiple layers of fabric, bound together by a staple (visible in the images in Fig. 3). The staple performed two functions: binding the upper layer of sample fabric to a lower layer which could be attached to other surfaces and handled without removing particles from the experiment, and, secondly, providing a landmark that could be used for image registration during the analysis of images from a persistence study.

## Methods

### Experiment 1: Implementing Manual Segmentation (Tracing)

Experiment one aimed to assess the reproducibility of manual segmentation (tracing) under optimal conditions, addressing the concerns raised over variation between examiners 12, 13, 14. Three participants were provided with three 300 × 300 pixel subsections of images in three rotations (Fig. 2), and asked to manually segment them to the best of their ability, by tracing over the fluorescent particles in white, and filling the background with black. The tracing was conducted in Adobe Photoshop (CS4) by opening the image, creating a new layer with a lowered opacity, and tracing over the foreground regions of the images using the brush and pencil tools. The participants were not aware that the nine images they were presented with were actually multiple orientations of three images. These images were all in focus and evenly illuminated; no efforts were made to deliberately complicate the task.

### Experiment 2: Implementing Manually Defined Threshold Values

In order to explore inter‐examiner reproducibility, images of ten swatches (Fig. 3) were given to 20 participants. The participants were asked to apply a threshold filter to segment the image into the foreground (fluorescent particles) and background (nonfluorescent fabric). This was done in Adobe Photoshop (CS4), using the pathway Image > Adjustments > Threshold. In order to explore intra‐examiner reproducibility, one examiner was asked to threshold each image 20 times with this method. This was conducted over a period of several weeks so that the examiner could not recall previous answers. The amount of time taken for each participant to complete the task was noted.

### Experiment 3: Implementing the Thresholding Algorithms

The thresholding algorithms were implemented using NIH ImageJ. ImageJ was chosen for its accessibility (it is freeware with an intuitive graphical user interface) and for its potential to be customised through the installation of additional plugins and the writing of macros 21, 22, 23, 24. In this study, a suite of 16 global thresholding algorithms were applied to the same 10 images used in experiment 2 (Fig. 3) utilising the plugin Auto Threshold v1.15 25. Mathematical notation for each algorithm is available in the publications outlined in 25, namely 26, 27, 28, 29, 30, 31, 32, 33, 34, 35, 36, 37, 38. Macros were written in order to open, process, and save the outputs of batches of images.

**Figure 4:**
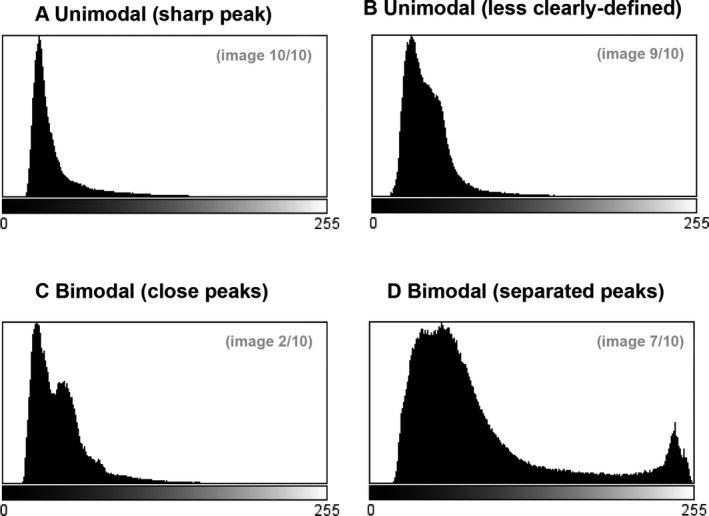
Examples of the four different histogram types observed in the images used in Experiments 2 and 3.

As it has been established within the fields of computer science and machine vision that some thresholding algorithms perform better or worse on unimodal or bimodal images, and with different levels of noise 8, it was considered important that the thresholding algorithms were tested upon images representative of all of the histogram shapes that one might encounter in a study involving fluorescent powder as a proxy for forensic trace evidence. Accordingly, the 10 images used in experiments 2 and 3 (Fig. 3) were representative of the four histogram types (Fig. 4) seen in the images generated by the persistence study from which the images were drawn (i.e., they were selected by stratified sampling from the available images).

### Evaluating the Accuracy of Segmented Outputs

In order to evaluate the accuracy of different segmentation methods, researchers tend to compare the output of each method to a “ground truth” generated by a human manually inspecting the image and defining the “true” segments (e.g., 12, 15, 16, 17). This is not, however, a “true” ground truth as it is to a certain extent subjective to the interpreter 15. Accordingly, here, instead of referring to a “ground truth,” the different segmentation outputs were compared against the “mean manual segmentation values (*n* = 3)” for each image. These were calculated by getting one examiner to “trace” each image used in experiments 2 and 3 three times. Three metrics (A–C) of the accuracy of analyses are presented here:

### Metric A: Absolute Error in the Percentage of the Image Identified as Foreground

The percentage of each image defined as foreground was calculated in ImageJ, using the pathway Analyse > Analyse Particles. While it would have been possible to assess the number of foreground regions identified, the percentage seemed a more appropriate measure since the number of regions is affected by contiguity (i.e., two contiguous particles would be counted as one region, and this study was primarily interested in the estimated extent of the foreground, not its distribution).

### Metric B: Relative Error in the Percentage of the Image Identified as Foreground

As well as expressing the error as an absolute value (i.e., the residual between the estimate of percentage foreground and the mean estimate of percentage foreground obtained by manual segmentation [tracing]), it is here also expressed as a relative value (i.e., the percentage overestimation or underestimation compared to the mean percentage value obtained from the manual segmentation). It was considered important to express both since the values for the extent of the foreground were variable, ranging from approximately 3% to approximately 22% of the image. Accordingly, a 1% absolute difference in the estimate is not equally significant for each image. Similarly, an apparently large relative deviation might translate to a relatively small absolute deviation.

### Metric C: The Dice Coefficient of Similarity

**Figure 5:**
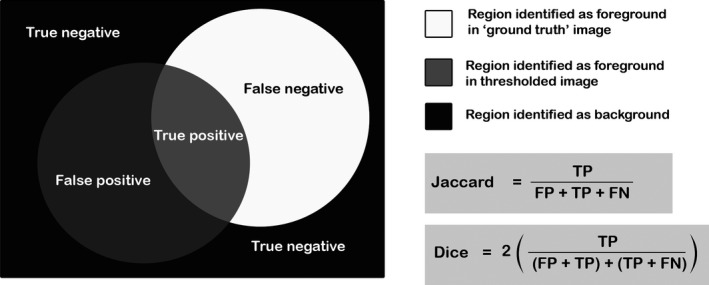
An explanation of the Dice and Jaccard Indices of similarity. FN, false negative; FP, false positive; TN, true negative; TP, true positive.

In order to ensure that the images had been segmented with fidelity, it was important to ensure that the segmented outputs were not just quantitatively similar (i.e., that, for example, both classified 5% of the pixels in the image as foreground) but also that they overlapped (i.e., that they identified the same 5% of the pixels as foreground). Accordingly, the Jaccard Index and Dice coefficients were calculated (two measures commonly used within quantitative comparison of segmented outputs 16). Both of these equations consider two images, asking whether each pixel is classified as foreground in either, both, or neither of the images (i.e., the area of the image which represents true and false positives and negatives, with 0 indicating no overlap between the images, and 1 indicating total overlap [Fig. 5]). This analysis was conducted in ImageJ using the LIM Tools Similarity Index plugin 39, 40. For experiment 1, the mean similarity index value was calculated by comparing images 1 and 2, 1 and 3, then 2 and 3, and taking the mean of the three values. For experiments 2 and 3, results were obtained by comparing the output image to the first manual segmentation output. Since both similarity coefficients produced similar outcomes, only one (the Dice coefficient) is presented in this paper, to illustrate the results.

## Results

### Experiment One: Evaluating the Reproducibility of Segmentation by Tracing

**Figure 6:**
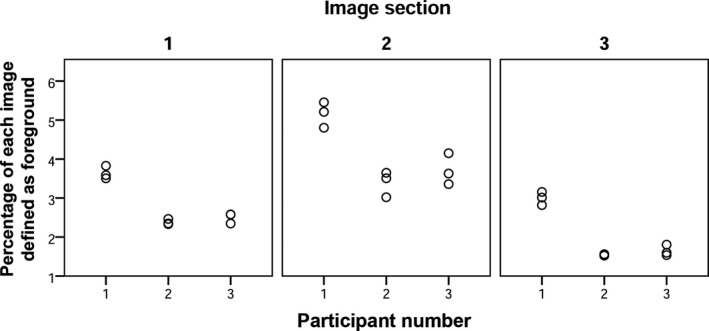
Variation in the percentage of each image section defined as foreground (*n* = 3) in Experiment 1.

Figure 6 displays the percentage of each 300 by 300 pixel image section defined as foreground by the three examiners (*n* = 3). Two trends were observed. Each examiner produced a range of values (i.e., there were similar but not identical outputs each time the image was segmented) and the mean value of these ranges varied between examiners (Table 2). An ANOVA was conducted on these data, and for all three of the images, this variation in the percentage of the image defined as foreground between the examiners was statistically significant at *p* < 0.01 (Table 2).

**Table 2:** Summary table for the percentage of each image defined as foreground

Image Section	Participant Number	*N*	Mean	SD	ANOVA Results, Comparing Participants		
					*f*‐ratio	*p*‐Value	*p* < 0.05?
1	1	3	3.64	0.17	84.817	0.00004	✓
	2	3	2.38	0.07			
	3	3	2.50	0.13			
2	1	3	5.16	0.33	20.986	0.00196	✓
	2	3	3.39	0.33			
	3	3	3.71	0.40			
3	1	3	3.00	0.17	122.184	0.00001	✓
	2	3	1.54	0.02			
	3	3	1.65	0.14			

Considering the Dice coefficient values produced when comparing each of the three segmentations for each examiner (i.e., comparing segmentations 1 and 2, 1 and 3, and 2 and 3 for each image section), the mean Dice coefficient value was 0.80 ± 0.12 across all three examiners (*n* = 27; Table 3). Examiner two yielded the highest coefficient values when comparing their three segmentation attempts, with a mean Dice Coefficient value across all images of 0.95 ± 0.02 (*n* = 9; Table 3). Examiners 1 and 3 exhibited similar levels of performance, with mean Dice Coefficient values of 0.71 ± 0.08 and 0.75 ± 0.02 respectively (*n* = 9), when comparing their three segmentation attempts.

**Table 3:** The mean Similarity Index values for the three examiners’ output images, when comparing segmentation attempts 1 and 2, 1 and 3, and 2 and 3

Participant Number	*N*	Dice Coefficient value			
		Mean	SD	Min	Max
1	9	0.71	0.08	0.56	0.77
2	9	0.95	0.02	0.90	0.97
3	9	0.75	0.02	0.71	0.79
Total	27	0.80	0.12		

### Experiment Two: Evaluating the Accuracy, Reproducibility, and Efficiency of Segmentation by a Manually Defined Threshold Value

#### Accuracy

**Figure 7:**
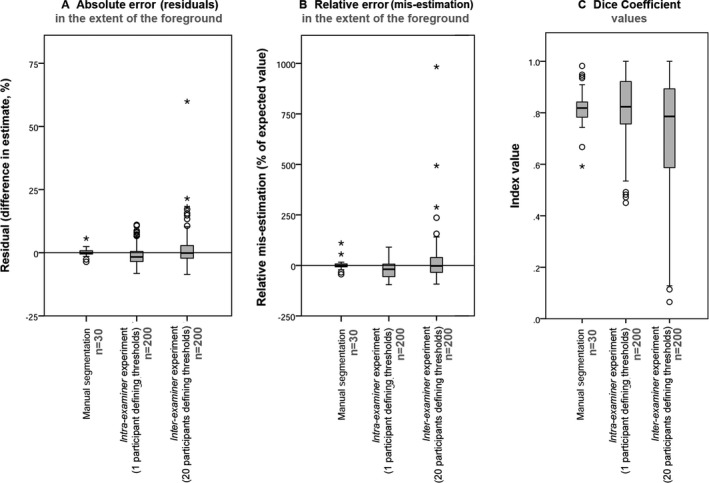
Metrics of performance for the manual methods of segmentation.

Segmentation by manually defining a threshold value appeared to offer good levels of accuracy when the individual runs were averaged; the mean values for the estimate of the extent of the foreground were within ±1% of the mean values obtained by tracing (Fig. 7 *A*; Table 4). The results from the intra‐examiner images (i.e., where one examiner had repeatedly manually defined a threshold value) tended to slightly underestimate the extent of the foreground, with a mean residual of −0.96 ± 3.73% (*n* = 200), which translated to a mean mis‐estimation of −21.06 ± 41.73% (*n* = 200; Fig. 7 *B*). The inter‐examiner images (i.e., where multiple examiners had manually defined a threshold value) tended to slightly overestimate the extent of the foreground, with a mean residual of 0.95 ± 6.45% (*n* = 200), and a mean overestimation of 11.50 ± 96.68% (*n* = 200) when compared to the mean values obtained by tracing (Fig. 7 *A*,*B*; Table 4).

**Table 4:** Descriptive statistics for Experiments 2 and 3

Segmentation method	Metrics of Performance								
	(A) Absolute Error (residuals) in the Extent of the Foreground (%)			(B) Relative Error (mis‐estimation) in the Extent of the Foreground (%)			(C) Dice Coefficient Values		
	*N*	Mean	SD	*N*	Mean	SD	*N*	Mean	SD
Manual									
Manual segmentation (tracing)	30	0.11	1.62	30	2.35	26.65	30	0.82	0.08
Manually defined threshold (1 examiner)	200	−0.96	3.73	200	−21.06	41.73	200	0.82	0.13
Manually defined threshold (20 examiners)	200	0.95	6.45	200	11.50	96.68	200	0.72	0.23
Global algorithms									
Default	10	3.84	7.74	10	70.48	144.25	10	0.62	0.30
Huang	10	25.44	21.11	10	567.20	594.79	10	0.33	0.32
Intermodes	10	−3.22	2.77	10	−51.82	36.62	10	0.74	0.22
IsoData	10	2.98	6.20	10	55.49	116.19	10	0.63	0.29
Li	10	9.61	11.37	10	159.87	211.10	10	0.54	0.34
MaxEntropy	10	−0.77	4.72	10	−22.40	51.96	10	0.79	0.16
Mean	10	24.26	9.41	10	466.74	329.82	10	0.32	0.30
MinError	10	53.74	20.58	10	917.85	565.52	10	0.20	0.20
Minimum	10	−5.09	2.69	10	−73.91	31.45	10	0.33	0.31
Moments	10	1.92	3.13	10	28.79	42.01	10	0.66	0.28
Otsu	10	1.44	3.17	10	25.42	51.20	10	0.64	0.28
Percentile	10	41.94	6.15	10	763.39	491.28	10	0.23	0.23
RenyiEntropy	10	0.18	5.08	10	−12.75	54.85	10	0.78	0.16
Shanbhag	10	−3.01	3.76	10	−52.57	46.90	10	0.55	0.31
Triange	10	4.04	3.24	10	51.82	41.91	10	0.59	0.25
Yen	10	0.58	5.69	10	−9.99	59.01	10	0.78	0.17

#### Reproducibility

The reproducibility of manually defining a threshold value was poorer than for manually segmenting the images (tracing). A much larger range and interquartile range of values for error were seen, along with multiple outliers (Fig. 7). The inter‐examiner images produced the widest interquartile range of errors and numerous extreme outliers, with a maximum overestimation of almost 1000% (Fig. 7 *B*). This range of estimates translated into a range of Similarity Index values; the minimum Dice coefficient values observed were 0.59 for tracing (*n* = 30), 0.45 for the intra‐examiner images (*n* = 200), and 0.07 (*n* = 200) for the inter‐examiner output images (Fig. 7 *C*). This is indicative of some segmentations of the images having very poor overlap with the supposedly accurate traced image.

#### Efficiency

Manually defining a threshold value required approximately one minute per image. The tracing, which was undertaken to create a mean against which the segmentations could be compared, took approximately one hour per image.

### Experiment Three: Evaluating the Performance of 16 Global Thresholding Algorithms

#### Accuracy

**Figure 8:**
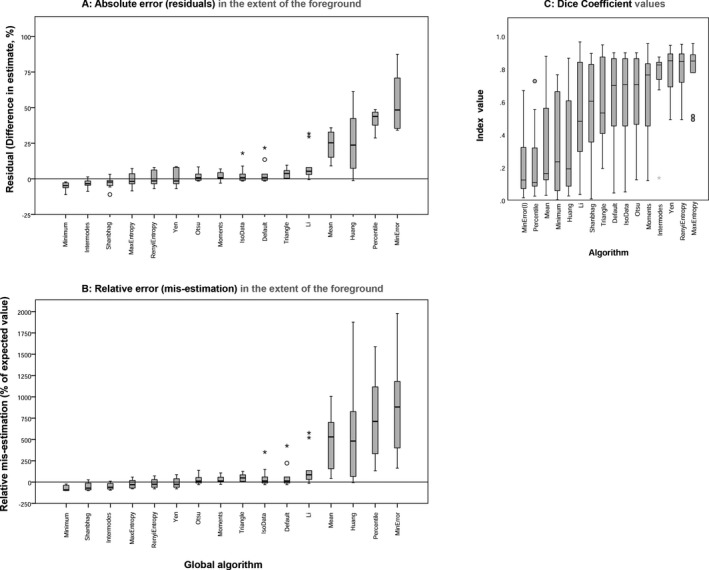
Metrics of performance for the global algorithms in order of ascending median.

**Figure 9:**
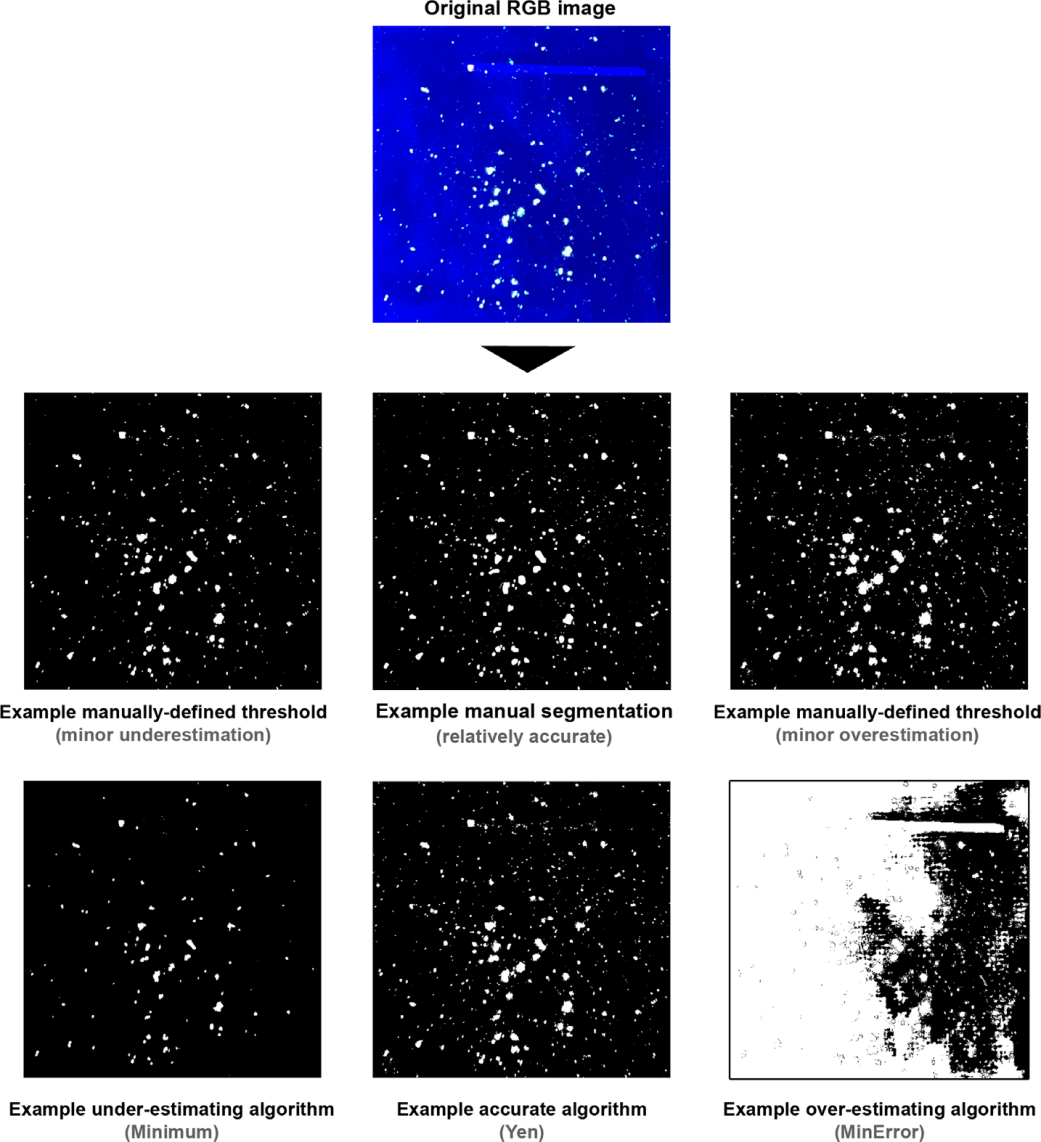
Example outputs for one image (image 1), showing varying levels of accuracy. [Color figure can be viewed at http://wileyonlinelibrary.com]

The global thresholding algorithms performed with varying levels of accuracy (Fig. 8; Table 4). While some of the algorithms vastly overestimated the extent of the foreground in comparison with the mean value obtained by tracing (e.g., MinError and Percentile [Fig. 9], which produced a mean estimate of the foreground of more than 500% of the expected values [Fig. 8B]), other algorithms resulted in estimates which were very similar to the mean manual segmentation values. The global algorithms which performed with greatest similarity to the mean manual segmentation values were Yen (mean residual 0.58 ± 5.69%, and a mean mis‐estimation of −9.99 ± 59.01% [*n* = 10]), MaxEntropy (mean residual −0.77 ± 4.72%, and a mean mis‐estimation of −22.40 ± 51.96% [*n* = 10]), and RenyiEntropy (mean residual 0.18 ± 5.08%, and a mean mis‐estimation of −12.75 ± 54.85% [*n* = 10]). The mean Dice coefficient values for these algorithms were comparable to those produced by the manual methods; 0.78 ± 0.17 for Yen, 0.79 ± 0.16 for MaxEntropy, and 0.78 ± 0.16 for RenyiEntropy (*n* = 10). All three algorithms produced a minimum Dice Coefficient value of 0.49 (*n* = 10). Comparing the three best‐performing algorithms to the manual methods (Table 4), it can be seen that the results produced by the algorithms are similar to the results produced by the manual methods.

#### Reproducibility

Global thresholding algorithms are deterministic, always producing the same output for a given input. The variation in accuracy seen in Fig. 8 corresponds to the algorithms performing with different levels of accuracy on the 10 different images upon which they were tested, when compared to the mean manual segmentation values for the extent of the foreground.

#### Efficiency

The algorithms, in combination with a macro, required less than 1‐second to open, process, save, and close each image.

## Discussion

### Experiment One: Evaluating the Reproducibility of Segmentation by Tracing

All three examiners produced ranges of values for their estimates of the extent of the foreground (i.e., variation was observed each time the image was traced). For all images, there was statistically significant variation between the results of the three examiners as to the percentage of the image that was defined as foreground. This is perhaps slightly surprising, as manual segmentation (tracing) is held to be the “gold standard” of segmentation techniques, against which others are compared 16. These findings support the assertions seen in the published literature that segmentation methods which involve manual input may be to a certain extent “observer‐dependent” 14 and variable between examiners when images contain regions of ambiguity (i.e., where pixels could be seen as belonging to either the foreground or the background), as in the analysis of images of soils 12.

It is worth emphasising that the images in experiment one were intended to represent optimal conditions for successful segmentation; no efforts were made to deliberately complicate the task of the examiners. The images were relatively small, taken with adequate and even illumination, and were in focus. It might be suggested that if such variation is observed under these conditions, it is possible that variation might be equal or greater for larger images taken under less standardised or less ideal lighting conditions or slightly out of focus. It also suggests that it may not be appropriate to divide the segmentation task of tracing for a single data set between multiple examiners.

Despite the variance between examiners, the similarity indices suggested that the internal reliability for each examiner was relatively good, with a minimum mean Dice coefficient value of 0.71 ± 0.08 (for examiner 1; *n* = 9), and a maximum mean Dice coefficient value of 0.95 ± 0.02 (for examiner 3). The difference in the Dice coefficient values between examiners suggests that some examiners may be more consistent when performing the task of manual segmentation than others. Given that, in the course of a larger experiment involving fluorescent particulates, it is likely that the segmentation would be carried out by a single examiner, it is therefore possible that the examiner would be unaware how much error the process of manual segmentation (tracing) might be introducing into the dataset.

### Experiment Two: Evaluating the Accuracy, Reproducibility, and Efficiency of Segmentation by a Manually Defined Threshold Value

In experiment two, it was seen that segmenting an image using a manually defined threshold level was far more efficient than tracing (taking approximately one minute per image, rather than one hour per image). Accordingly, while it would not be feasible to trace every image in a large dataset, it might be possible to segment them by manually defining threshold values.

While manually defining a threshold level offered, on average, a similar level of accuracy to manual segmentation (tracing), it offered a far lower level of reproducibility. While the mean values returned for the estimates of the foreground were within ±1% of the mean traced estimates, large ranges of values were seen for the estimates, and numerous outliers were observed (Fig. 7). Therefore, while it was far faster than manually segmenting (tracing) the images, manually defining a threshold level was seen to introduce a larger amount of error into the dataset.

Since the experiments conducted here suggested that manually defining a threshold value yielded results that were highly variable (i.e., it is possible that the segmented output will accurately reflect the input, but it is plausible that it will not), it might be suggested that manually defining a threshold level might not be a reliable method of segmentation for this form of ultraviolet fluorescence imagery. Since the quality of any quantitative output is contingent upon the quality of the segmentation step, this may be considered problematic.

### Experiment Three: Evaluating the Performance of 16 Global Thresholding Algorithms

The global algorithms tested here offered reproducible estimates for the extent of the foreground (always returning the same output from the same input), with efficiency (taking less than 1‐second to open, process, save, and close an image). The different algorithms performed with varying levels of accuracy (Fig. 8). The best‐performing algorithms, which in these experiments were the Yen, MaxEntropy, and RenyiEntropy global algorithms, offered mean estimates of the extent of the foreground that were concordant with (i.e., within ±1% of) the mean traced estimates (Fig. 8; Table 4).

Accordingly, on the basis of maximising reproducibility (without sacrificing accuracy), for a robust image processing workflow which is capable of processing large numbers of ultraviolet fluorescence images, these experiments suggest that it might be advisable to employ an appropriate algorithm. For future work, using an appropriate algorithm may provide a fast method of image segmentation, which does not compromise on levels of accuracy or reliability. The results of these experiments suggest that for ultraviolet fluorescence imagery of green powder fluorescing on a dark fabric background, the Yen, RenyiEntropy, or MaxEntropy algorithms may be appropriate. It should be noted, however, that since thresholding algorithms could perform with different levels of accuracy on images with different amounts and distributions of fluorescent material, it would be advisable for researchers to perform an evaluative study on the specific images to be used before employing a particular algorithm within a transfer and persistence experiment.

Future work could consider further testing of these algorithms under less ideal conditions (e.g., with less homogeneous lighting, out of focus images, or where there is less contrast between the foreground and background of the image). It is worth stressing that the algorithms tested here (i.e., global thresholding algorithms) are amongst the simplest methods of image segmentation; future work may want to explore the performance of more sophisticated segmentation methods within this context.

In summary, for images of fluorescent powder on dark fabric under ultraviolet illumination, manual segmentation (tracing) offered high levels of accuracy when compared to the mean manual segmentation and moderate reproducibility, but lacked efficiency, taking too long to be employed in a much larger study. Manual thresholding offered similar levels of accuracy and improved efficiency, but sacrificed reproducibility. A thresholding algorithm can offer improved reproducibility, superior efficiency, and, if the algorithm is appropriate, it can do so without sacrificing accuracy.

## Conclusions

Four main conclusions can be drawn from these experiments.

1. When manually segmenting (tracing) images of fluorescent powder under ultraviolet illumination, different examiners may arrive at different answers as to the extent of the foreground, and variation may be observed when the same examiner repeatedly segments an image.
2. While segmentation by manually applying a threshold can offer a faster alternative to manual segmentation (tracing), this method also raises problems regarding the reproducibility of results. While a mean value may approach an accurate answer for quantitative analyses, a single segmentation may produce an inaccurate value (i.e., in these experiments a range of values and outliers were seen).
3. Using an algorithm to define a threshold value can overcome these issues around reproducibility while offering increased efficiency. In these experiments, it was suggested that many algorithms are inappropriate for use with ultraviolet fluorescence imagery, but some offer results which are quantitatively similar to the outputs achieved by manual methods.
4. In these experiments, the three algorithms which performed with the greatest accuracy were Yen, MaxEntropy, and RenyiEntropy. All three of these global algorithms produced results which mis‐estimated the foreground by amounts comparable to a human examiner conducting manual segmentation, but at much faster speeds. It may be possible to employ these algorithms in future experiments as part of a rapid and robust image processing workflow.

Together, these findings suggest that persistence studies which employ fluorescent powder proxies for trace evidence could harness the power of thresholding algorithms and macros to accelerate data analysis without compromising on accuracy. Establishing a robust automated approach for segmentation has the potential to significantly expand the amount of data that can be collected regarding the transfer and persistence of trace particles. Such data can provide a valuable empirical evidence base to underpin the interpretation of forensic trace evidence and to respond to calls for further experimentation within these areas 6, 41, 42.
